# Efficacy and safety of acupuncture combined with Chinese Herbal Medicine for diabetic nephropathy: A protocol for systematic review and meta-analysis

**DOI:** 10.1097/MD.0000000000027087

**Published:** 2021-09-03

**Authors:** Ziyang Yu, Wenfeng Zhang, Borui Li, Pengjie Bao, Fengyang Wang, Jian Sun, Guojiao Song, Lu Yin, Zheng Nan

**Affiliations:** a Internal Medicine of Traditional Chinese Medicine; b Formulas of Chinese Medicine, Changchun University of Chinese Medicine; c Internal Medicine of Traditional Chinese Medicine, Changchun Traditional Chinese Medicine Hospital; d Department of Acupuncture and Tuina, Changchun University of Chinese Medicine; e Gynecology of Chinese Medicine, Traditional Chinese Medicine Hospital of Jilin Province; f Gynecology of Chinese Medicine, Traditional Medical Hospital of Changchun University of Chinese Medicine; g Internal Medicine of Traditional Chinese Medicine, Traditional Chinese Medicine Hospital of Jilin Province, Changchun, China.

**Keywords:** acupuncture, Chinese herbal medicine, diabetic nephropathy, effectiveness, protocol, safety, systematic review, traditional Chinese medicine

## Abstract

**Background:**

Diabetic nephropathy (DN) is one of the most serious complications in the development of diabetes mellitus, which has become the main cause of end-stage renal disease and one of the main causes of death in diabetic patients. With the prevalence of diabetes, the number of patients at risk for developing DN is increasing, with 20–40 percent of all patients with diabetes at risk for developing DN. Acupuncture and Chinese herbal medicine treatments are often combined to treat DN; however, there has been no meta-analysis on their synergistic effects. Therefore, we aimed to perform a systematic review and meta-analysis to estimate the effectiveness of acupuncture combined with Chinese herbal medicine for DN treatment.

**Methods:**

Nine electronic databases were retrieved for this study. The English databases mainly retrieved PubMed, Web of Science, Embase, AMED, and the Cochrane Library, while the CNKI, VIP, CBM, and Wanfang databases were used to retrieve the Chinese literature. There is no definite time limit for the retrieval literature, and the languages are limited to Chinese and English. We will consider articles published between database initiation and August 2021. We used Review Manager 5.4, provided by the Cochrane Collaborative Network for statistical analysis. Clinical randomized controlled trials related to acupuncture combined with Chinese herbal medicine for DN were included in this study. Research selection, data extraction, and research quality assessments were independently completed by two researchers. We then assessed the quality and risk of the included studies and observed the outcome measures.

**Results:**

This study provides a high-quality synthesis to assess the effectiveness and safety of acupuncture combined with Chinese herbal medicine for treating DN.

**Conclusion:**

This systematic review will provide evidence to determine whether acupuncture combined with Chinese herbal medicine is an effective and safe intervention for patients with DN.

**Ethics and dissemination:**

The protocol of the systematic review does not require ethical approval because it does not involve humans. This article will be published in peer-reviewed journals and presented at relevant conferences.

**Registration number:**

INPLASY202180018

## 1 Introduction

Diabetic nephropathy (DN) is the most common cause of end-stage renal disease (ESRD).^[1,2]^ It is characterized by proteinuria, decline in glomerular filtration, hypertension, and a high risk of cardiovascular morbidity and mortality.^[3–5]^ As the epidemic of diabetes spreads, the number of patients at risk for developing DN is increasing, which occurs in 20% to 40% of all diabetic patients.^[6,7]^ According to an epidemiological statistical report, the number of diabetic patients worldwide will reach 366 million in 2030, while the number of DN patients will exceed 100 million.^[8]^ Family and society have brought huge economic burdens, but patients and their families have also brought greater psychological pressure.^[9,10]^

The pathogenesis of diabetic nephropathy is complex. Although strict control of blood glucose can delay the progression of the disease, the current treatment effect is still not ideal.^[11–13]^ Therefore, it is very important to study the exact pathogenesis of the disease to determine the prognosis of patients. Hemodynamic and metabolic factors are the main causes of DN, and other risk factors such as advanced glycation end product (AGE) and oxidative stress (OS) are also believed to be involved in the pathogenesis of diabetes and its related complications.^[14–16]^ In recent years, acupuncture combined with Chinese herbal medicine (CHM) has been widely used in the treatment of diabetes and its complications, and has many advantages over conventional medical approaches in the prevention of diabetic complications.^[17]^ Therefore, this study aimed to conduct a meta-analysis of acupuncture combined with Chinese herbal medicine for the treatment of DN to clarify its efficacy.

## 2 Methods and analysis

This systematic review protocol was registered in Inplasy (INPLASY202180018). (https://inplasy.com/inplasy-2021-8-0018/)The systematic review will be performed following the guidelines of the Preferred Reporting Items for Systematic Review and Meta-Analysis Protocols (PRISMA-P) 2015.^[18]^

### 2.1 Inclusion criteria

#### 2.1.1 Types of participants

Participants who were definitively diagnosed with DN were included. No limitations of location, educational background, and gender were imposed.

#### 2.1.2 Types of studies

This study will only consider randomized controlled trials (RCTs) of acupuncture combined with Chinese herbal medicine for the treatment of patients with DN. However, other studies, such as animal studies, reviews, case studies, non-controlled studies, and quasi-RCTs, were excluded.

#### 2.1.3 Types of interventions

The intervention included acupuncture and Chinese herbal medicine. The control intervention included simple Western medicine, such as placebo or ACEI/ARB. Hypoglycemic therapy was used as a co-intervention in both arms, including oral hypoglycemic drugs, insulin, and exercise, or did not receive any treatment as a blank control.

#### 2.1.4 Types of outcomes

The primary outcomes included 24-hour urine protein quantitation, urinary albumin excretion rate, glomerular filtration rate, and fasting blood glucose level. The secondary outcome measure was based on TCM syndrome evaluation criteria.

##### 2.1.4.1 Healing

The clinical symptoms and signs of TCM disappeared or almost disappeared, and the syndrome score was reduced by ≥90%; urine protein excretion rate and creatinine clearance rate all returned to normal.

The clinical symptoms and signs of TCM were obviously improved, and the syndrome score was reduced by ≥60% and < 90%; urine protein excretion rate decreased by ≥50% and < 70%; creatinine clearance was normal.

##### 2.1.4.2 Effeciency

Chinese medicine clinical symptoms and signs improved, syndrome scores decreased by < 60%, but ≥30%; urinary protein excretion rate decreased by ≥20% and < 50%; creatinine clearance was normal. Invalid: The clinical symptoms and signs of TCM did not improve or worsen, and the syndrome score was reduced by < 30%. Integral variation formula (nimodipine method: [(pretreatment score – post-treatment score) pretreatment score] 100%.

### 2.2 Data sources and search methods

#### 2.2.1 Electronic searches

This study will use the Cochrane Library, Web of Science, PubMed, Embase, Allied and Complementary Medicine Database (AMED), China Biomedical Literature Database (CBM), China National Knowledge Infrastructure (CNKI), China Science and Technology Journal Database (VIP), Wanfang Database, and Ongoing Clinical Trials Database. There is no definite time limit for the retrieval literature, and the languages are limited to Chinese and English. We will consider articles published between database initiation and August 2021. The search terms were acupuncture, needling, Chinese herbal medicine, Chinese medicine, traditional Chinese medicine, proprietary Chinese medicine, and diabetic nephropathy. The search strategy for the PUBMED is presented in Table 1. Similar research strategies have been adopted for other electronic databases.

**Table 1:** . Search strategy for the PubMed database.

Number	Terms
#1	Diabetic nephropathy (all field)
#2	Diabetic kidney disease (all field)
#3	Diabetic (all field)
#4	Nephropathy (all field)
#5	#1 OR #2–4
#6	Acupuncture (all field)
#7	Needling (all field)
#8	Acupoint (all field)
#9	Acupuncture treatment (all field)
#10	Scalp acupuncture (all field)
#11	Fire needling (all field)
#12	Ear acupuncture (all field)
#13	Intradermal needling (all field)
#14	Auricular acupuncture (all field)
#15	Electroacupuncture (all field)
#16	Catgut embedding (all field)
#17	#6 OR #7–16
#18	Chinese medicine (all field)
#19	Traditional Chinese medicine (all field)
#20	Chinese herb medicine (all field)
#21	Proprietary Chinese medicine (all field)
#22	Chinese Herbs (all field)
#23	Chinese herbal (all field)
#24	#18 OR #19–23
#25	randomized controlled trial (all field)
#26	randomly (all field)
#27	controlled clinical trial (all field)
#28	randomized (all field)
#29	random allocation (all field)
#30	placebo (all field)
#31	single-blind method (all field)
#32	double-blind method (all field)
#33	trials (all field)
#34	Comparators
#35	Allocation
#36	#25 OR #26–35
#37	#5 And #17 And #24 And #36

#### 2.2.2 Searching for other resources

To avoid missing any other relevant studies, we will also search other source records, including conference proceedings, clinical registries, and reference lists of relevant reviews.

### 2.3 Data selection

**Figure 1:**
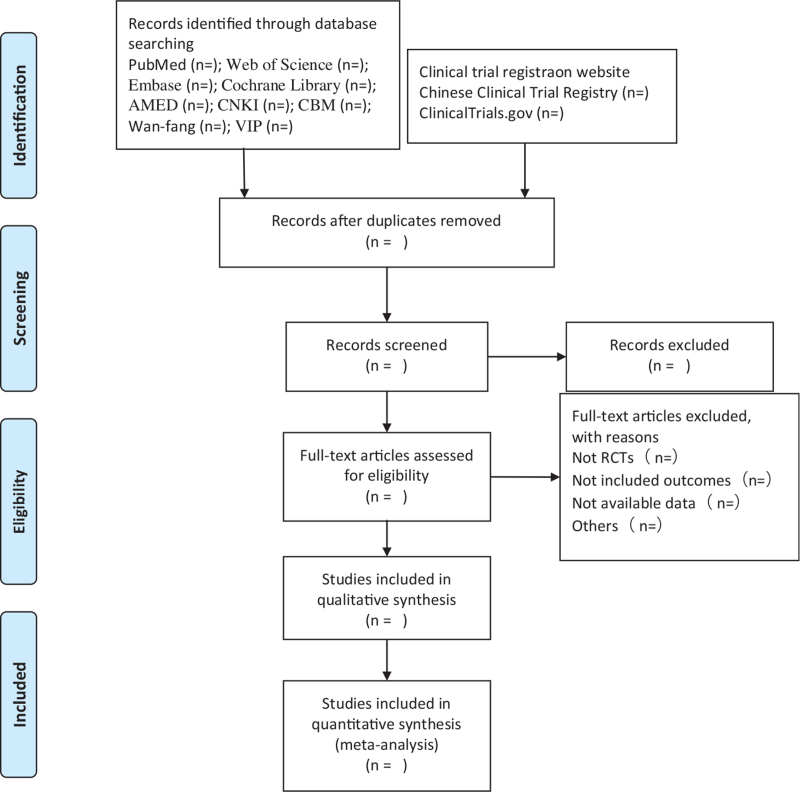
Flow diagram of study selection process.

First, two investigators used Endnote X9 software to conduct a preliminary assessment of the title and abstract of each document in the database based on the established criteria for inclusion in the study to select eligible studies. After a preliminary assessment, the full text of the selected literature was evaluated, and the uncontrolled study, no randomization, inconsistent evaluation criteria, and similar data were excluded. Finally, the final included literature was exchanged and checked by researchers. If the two researchers disagree on the results of a study or eventual inclusion, we will resolve it through discussion or consultation with a third person. A flowchart of the screening process is presented in Figure 1.

### 2.4 Data extraction

Before data collection, the study team built a data extraction sheet. Two authors separately collected relevant information from each eligible study. The data extraction table mainly includes the following contents: research title, first author, year of publication, sample size, duration of disease, intervention measures, outcome indicators, adverse reactions, and so on. If a study has unclear or inadequate information, we will attempt to contact the authors via email. Still, we may consider excluding the study if we cannot find the relevant information in various ways.

### 2.5 Risk of bias assessment

Two investigators will separately assess the risk of bias of the selected RCTs using the Cochrane risk of bias assessment tool. The evaluation of each study mainly included the following seven aspects: random sequence generation, allocation hiding, blinding of participants and personnel, blinding of outcome assessment, incomplete outcome data, incomplete outcome data, selective outcome reporting, and other biases. Finally, the bias of the study will be rated on three levels: “low”, “high”, and “ambiguous”. These even domains will be separately appraised by two reviews, and discrepancies will be addressed by consulting a third reviewer.

### 2.6 Data synthesis

In this study, we will apply RevMan 5.4 software for statistical analysis. The risk ratio and 95% confidence intervals (CIs) were collected for enumeration data, while the mean difference or standardized mean difference and 95% CIs were used to calculate continuous outcome data. The heterogeneity of the data was tested by calculating I^2^ statistics. The study was not considered to have a large heterogeneity when the I^2^ value was less than 50%. when the I^2^ value exceeded 50%, there was significant statistical heterogeneity among the trials. When there is homogeneity in the merged outcome results across sufficient studies, a meta-analysis will be conducted. Otherwise, we performed a subgroup analysis to explore the causes of the heterogeneity.

### 2.7 Subgroup analysis

We will investigate the source of heterogeneity using subgroup analysis based on different interventions, controls, and outcomes.

### 2.8 Assessment of reporting biases

Funnel plot^[19]^ and Egger regression test^[20]^ were used to determine potential reporting bias if sufficient studies were included.

### 2.9 Sensitivity analysis

We will carry out a sensitivity analysis to investigate the robustness and stability of outcome results by removing low methodological quality studies. The main analysis points included the impact of method quality, sample size, and missing data on the study. In this way, we will be able to assess the impact of individual studies on the overall results and determine whether the results are strong.

### 2.10 Grading the quality of evidence

For the quality evaluation of the whole study, we used the grading method of “recommended Evaluation, Development and Evaluation (Grade) Guide”. It was evaluated according to the five aspects of the study: limitations, inconsistencies, indirectness, inaccuracy, and publication bias of the research design. In the end, the quality of the research will be divided into 4 levels from high to low are high, medium, low, and very low.

### 2.11 Ethics and dissemination

No ethical approval is required because this study was based on data from the published literature. This study is expected to be published in a peer-reviewed journal.

## 3 Discussion

Diabetic nephropathy (DN) is a major microvascular complication of diabetes. Most patients present with latent nephropathy at an early stage, develop nephrotic syndrome, and finally enter end-stage DN, which is a common cause of end-stage renal disease.^[21]^ The clinical symptoms of diabetic nephropathy are relatively invisible. Renal damage in patients with proteinuria has entered a crisis stage, and the prognosis of diabetic nephropathy patients cannot be underestimated.^[22]^ In the application of traditional Chinese medicine in the treatment of diabetic nephropathy, it can control blood pressure and reduce blood sugar, reduce proteinuria to play the role of protect the kidney, and delay the occurrence and development of diabetic nephropathy, which fully reflects the ideal application prospects of traditional Chinese medicine. Acupuncture is an important part of complementary and alternative medicine in Western countries and has been used for thousands of years in traditional Chinese medicine.^[23]^ Acupuncture and acupuncture-like somatic nerve stimulation have also been successfully used to treat different kidney diseases and related complications.^[24]^ This study will provide helpful evidence of acupuncture combined with Chinese herbal medicine for the treatment of DN at evidence-based medicine levels. The findings of this study will also affect clinical practice and health-related policies to improve DN treatment approaches.

## Author contributions

**Conceptualization:** Ziyang Yu, Zheng Nan.

**Data curation:** Ziyang Yu, Wenfeng Zhang.

**Formal analysis:** Borui LI, Pengjie Bao.

**Funding acquisition:** Zheng Nan.

**Investigation:** Fengyang Wang, Jian Sun.

**Supervision:** Guojiao Song, Lu Yin.
